# Spatiotemporal Characteristics of Event-Related Potentials Triggered by Unexpected Events during Simulated Driving and Influence of Vigilance

**DOI:** 10.3390/s21217274

**Published:** 2021-11-01

**Authors:** Pukyeong Seo, Hyun Kim, Kyung Hwan Kim

**Affiliations:** Department of Biomedical Engineering, College of Health Science, Yonsei University, 1, Yeonsedae-gil, Heungeop-myeon, Wonju-si 26493, Gangwon-do, Korea; seopk@yonsei.ac.kr (P.S.); dosteps@yonsei.ac.kr (H.K.)

**Keywords:** electroencephalogram, event-related potential, cortical current source density, driving simulator, vigilance

## Abstract

We investigated the spatiotemporal characteristics of brain activity due to sudden events during monotonous driving and how it changes with vigilance level. Two types of sudden events, emergency stop and car drifting, were presented using driving simulator, and event-related potentials (ERPs) were measured. From the ERPs of both types of events, an early component representing sensory information processing and a late component were observed. The early component was expected to represent sensory information processing, which corresponded to visual and somatosensory/vestibular information processing for the sudden stop and lane departure tasks, respectively. The late components showed spatiotemporal characteristics of the well-known P300 component for both types of events. Common characteristic brain activities occurred in response to sudden events, regardless of the type. The modulation of brain activity due to the vigilance level also shared common characteristics between the two types. We expect that our results will contribute to the development of an effective means to assist drivers’ reactions to ambulatory situations.

## 1. Introduction

Driving is a complicated task that involves a significant amount of cognitive information processing by the brain. It is important to understand brain activities in response to sudden, unexpected events during driving, as it may provide an effective means to assist drivers’ reactions to ambulatory situations. Based on this, an effective warning scheme and automatic driving assistant system may be devised to enable prompt reactions to sudden events. The driver’s capability to respond to unexpected situations should also be monitored. It has been reported that electroencephalograms (EEGs) can be used successfully to estimate attention, sleepiness, and drowsiness [1,2,3], which results in a decline in cognitive function for the reaction to sudden events.

In this study, we attempted to reveal spatiotemporal characteristics of brain activity due to sudden events during driving, and the influence of vigilance level on them. We monitored event-related potentials (ERPs) triggered by two types of sudden events that frequently occur during driving: emergency stops and car drifting. These two types of events commonly trigger a series of cognitive processes, including sensory perception, attentional target recognition, and response generation. The most significant difference between the two types lies in the sensory modality for the perception of emergency, whereas the later steps of the cognitive process should be shared between the two types. The ERP components corresponding to each cognitive process were analyzed along with their cortical current sources. Similarities and differences between the two types of tasks were identified in terms of spatiotemporal characteristics and the influence of vigilance level on them.

The contribution of this study is to reveal the brain activities in response to sudden emergency events during driving, which has not been fully identified so far. Especially, the neural activities triggered by two different sensory modalities, i.e., visual and somatosensory stimuli, were compared. Thus, we found that the brain activities of the two sensory modalities share similar temporal characteristics when processing information on unexpected events.

## 2. Methods

### 2.1. Participants

Twenty university students without neuropsychiatric diseases participated in the study (23.9 ± 1.4 years, 18 men, 2 women). All subjects had normal or corrected-to-normal vision and had steady driving experience of more than 5 years (5.1 ± 1.4 years), without any car accidents within the past 2 years. They had adequate sleep (longer than 8 h) during the night before the experiment, and did not consume caffeine, nicotine, or other drugs on the day of the experiment. This study was approved by Yonsei University Wonju Institutional Review Board (IRB 1041849-201709-BM-095-01). Written informed consent was obtained from each subject prior to the experiment.

### 2.2. Simulated Driving and Experimental Procedure

A driving simulation environment was constructed using a PC-based steering wheel and pedal set (G29, Logitech, Lausanne, Switzerland) and three 24-inch thin bezel monitors (FHD240Slim, Wasabimango, Republic of Korea). The simulation software was developed on the basis of a 3D game creation platform, Unreal Engine 4 (Epic Games, NC, USA).

**Figure 1:**
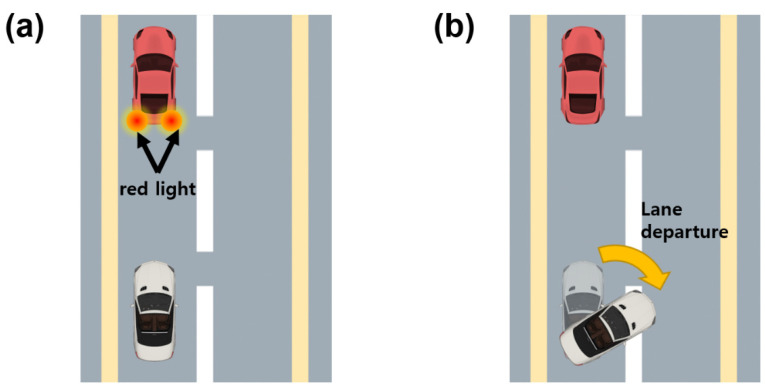
Schematic diagram of two types of unexpected situations. Participants always drive white cars on two-lane roads along with red cars ahead. (**a**) Sudden stop; (**b**) Lane departure.

The virtual driving environment consisted of a simple two–lane straight road and one vehicle moving in front. The subjects were instructed to drive in the left lane while maintaining a safe distance from the car in front for 90 min. In addition, they were instructed to respond as soon as possible when unexpected events occurred. Two types of unexpected events are shown in Figure 1. In the first type, a sudden stop, the car in front stopped suddenly (Figure 1a). The second type was lane departure, in which the driver’s vehicle suddenly slipped to the right and departed from the driving lane against the driver’s intention (Figure 1b). Both types of events were presented 90 times each with a randomized interstimulus interval (mean: 30 s, SD: 1 s).

The experiment was conducted for 90 min from 20:00, after normal daytime activities of typical college students, when the fatigue from their daily activities had accumulated. To understand the instructions and become familiar with the experimental environment, the subjects engaged in a 1-h practice session before the main experiment.

### 2.3. Driving Performance Analysis Based on Behavioral Response

Response time (RT) was measured to evaluate driving performance, which can be interpreted in relation to vigilance or attention level. For the lane departure task, the RT was measured from the onset of the vehicle drifting to the onset of the steering wheel being turned by the driver, to return to the original lane position (Figure 1a). The RT of the sudden stop task was measured from the turn-on time of the brake light of the car in front, to the brake pedal press by the driver (Figure 1b).

The vigilance of each trial was classified as either ‘High’ or ‘Low’ according to the RT, by comparing to the averaged RT for each subject. That is, trials with an RT shorter than the average were labeled as High, and those with RT longer than the average were labeled as Low. Only trials with correct responses with RTs of 300–2000 ms for sudden stop and 300–1500 ms for lane departure were used for further analysis. The number of trials per subject included in the analysis was 64.50 ± 13.82 for sudden stop, and 71.35 ± 9.00 for lane departure.

### 2.4. EEG Recording and Analysis

EEGs were recorded at a sampling rate of 500 Hz using a 64 channel Ag/AgCl electrode cap (actiCAP; Brain Products, GmbH, Munich, Germany) according to the extended 10–10 system. The impedances of all electrodes were kept below 50 kΩ. The reference and ground electrodes were placed at FCz and AFz, respectively. A bandpass filter (0.03–100 Hz) and 60 Hz notch filter were applied. EEGLAB was used to preprocess the recorded EEG signals [4].

The EEG waveforms were excluded from further analysis if they were severely contaminated by drift or transient artifacts due to head/electrode motion. The recorded EEG signals were segmented into single-trial waveforms of 1200 ms duration (from −200 ms to 1000 ms post-stimulus). The baseline of each trial was corrected using a pre-stimulus waveform (−200 ms to 0 ms). Stereotyped artifacts, such as eye movements or EMG, were removed using independent component analysis [5]. Subsequently, the waveforms were referenced according to an average reference. The averaged ERP waveforms were calculated for High and Low conditions within −200 to 1000 ms time intervals. A 20 Hz low-pass filter was applied to the averaged ERP waveforms.

### 2.5. Cortical Current Source Reconstruction

We used an open-source MATLAB toolbox, Brainstorm, to calculate the current source density on the cortical surface, and thus the event-related current density (ERCD) time series could be obtained. The forward problem was solved using the boundary element method (BEM) [6] based on default anatomy using the International Consortium for Brain Mapping (ICBM) 152 template [7]. OpenMEEG in the Brainstorm toolbox was used to compute the head model with a symmetric BEM. The number of vertices in the scalp, outer skull, and inner skull layers was set to 1922, and the conductivities were set to 1, 0.0125, and 1, respectively. The thickness of the skull was set at 4 mm. The current densities at 15,002 vertices on the cortical surface were estimated by solving the inverse problem with a weighted minimum norm estimation [8]. All vertices on the cortical surface were grouped into 62 functionally similar regions, based on the Desikan-Killiany atlas [9].

## 3. Results

### 3.1. Response Time

The average RT for the sudden stop task was 1192.4 ± 186.8 ms. The average RT for High (30.9 ± 6.8 trials) was 1030.1 ± 152.1 ms, and for Low (30.5 ± 6.8 trials), 1404.1 ± 212.8 ms on average, showing a significant difference (t = 13.8, *p* < 0.001). The average RT for the lane departure task was 714.3 ± 88.2 ms. The average RTs were also significantly different between the vigilance conditions (t = 12.5, *p* < 0.001, High: 614.7 ± 53.6 ms, Low: 872.1 ± 136.4 ms).

### 3.2. Characterization of ERP Components and ERCD

#### 3.2.1. Sudden Stop Task

**Figure 2:**
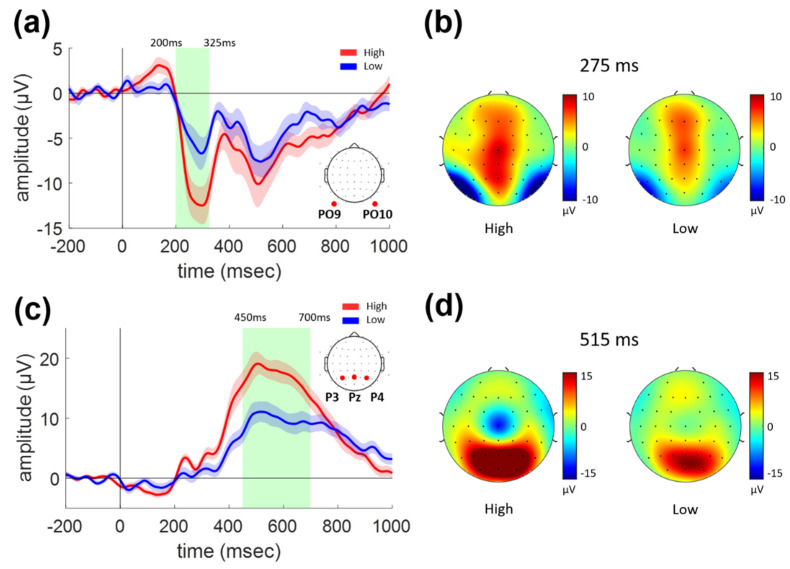
The grand-average ERP waveforms and topographical distributions for sudden stop task. (**a**) The grand-average ERP waveforms for PO9 and PO10 electrodes. (**b**) Topographical distributions of ERP at 275 ms. (**c**) The grand-average ERP waveforms for P3, Pz, and P4 electrodes. (**d**) Topographical distributions of ERP at 515 ms.

Two dominant ERP components were found at 200–325 ms and 450–700 ms for the sudden stop task. Figure 2 shows the grand-average ERP waveforms and topographical distributions for the sudden stop task. The waveform of the early component in the bilateral parieto-occipital region is shown in Figure 2a as the average of waveforms at the PO9 and PO10 electrodes where the topographical peak was located, as shown in Figure 2b. The green shading in Figure 2a denotes the temporal range when a significant difference between the High and Low conditions was manifested. The average ERP amplitude was significantly decreased, at 200–325 ms for the Low as compared to the High (paired *t*-test, *p* < 0.001).

A positive ERP component with a broad peak was observed at 450–700 ms over the widespread parieto-occipital region, as shown in Figure 2c,d. The average ERP amplitude was significantly decreased in the Low condition as compared to the High (paired *t*-test, *p* < 0.001).

**Figure 3:**
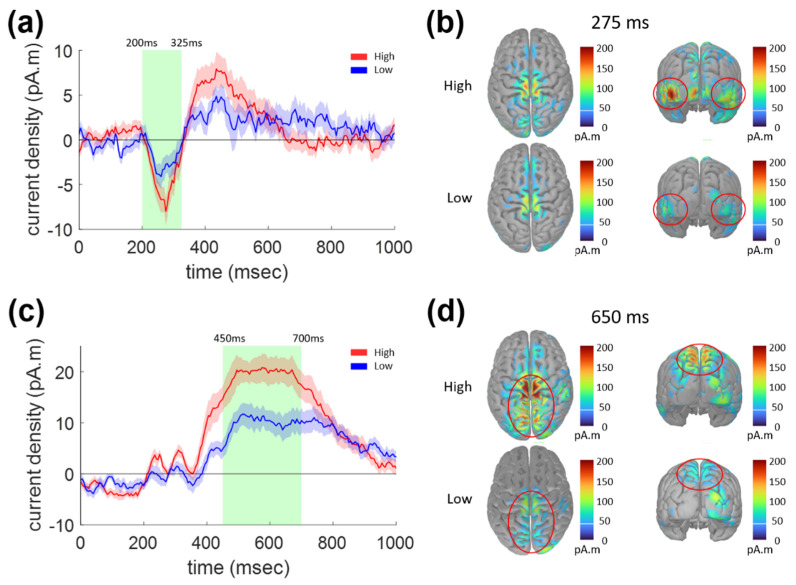
ERCD waveforms for the sudden stop task (0–1000 ms) and the distribution of current source density on cortical surface. (**a**) ERCD waveforms averaged over the vertices belonging to lateral occipital regions. (**b**) Distribution of ERCD on cortical surface at 275 ms. (**c**) ERCD waveforms averaged over the vertices belonging to superior parietal regions. (**d**) Distribution of ERCD on cortical surface at 650 ms.

These ERP components can be attributed to the peaks in the estimated cortical current source time series. Figure 3 shows ERCD waveforms for the sudden stop task (0–1000 ms) and the distribution of the current source density on the cortical surface. Figure 3a shows the ERCD waveforms averaged over the vertices belonging to the lateral occipital regions in the Desikan-Killiany atlas. Figure 3b shows the ERCD distribution at 275 ms, with peak activities within the left occipital (LO) and right occipital (RO) regions (indicated by red circles). Similar to the first ERP component shown in Figure 2a, the ERCD in the LO/RO regions was significantly different between the High and Low conditions in the 200–325 ms epoch (paired *t*-test, *p* = 0.024). 

Figure 3c shows the ERCD waveforms averaged over the vertices within the left and right superior parietal (LSP and RSP) regions in the Desikan-Killiany atlas. These waveforms were significantly different between the High and Low conditions at 450–700 ms (paired *t*-test, *p* < 0.001). The distribution of the ERCD at 650 ms is shown in Figure 3d, which shows that strong activities were focused around the central sulcus and superior parietal region.

#### 3.2.2. Lane Departure Task

**Figure 4:**
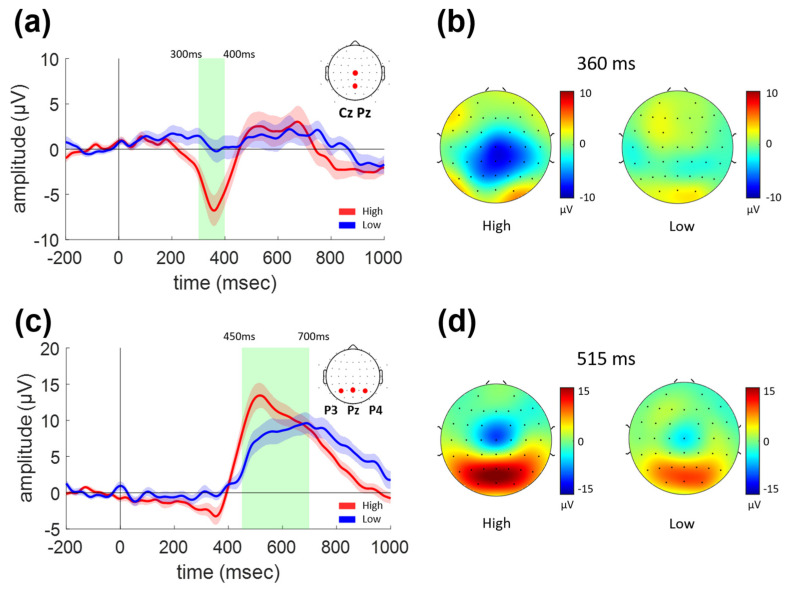
The grand-average ERP waveforms and topographical distributions for lane departure task. (**a**) The grand-average ERP waveforms for Cz and Pz electrodes. (**b**) Topographical distributions of ERP at 360 ms. (**c**) The grand-average ERP waveforms for P3, Pz, and P4 electrodes. (**d**) Topographical distributions of ERP at 515 ms.

Two major ERP components were also identified for the lane departure task, at 300–400 ms and 450–700 ms, as shown in Figure 4. Figure 4a,b shows the waveforms of the first ERP components (average of Cz and Pz) and their topographies at 360 ms, respectively. An apparent negative peak was observed in the central and parietal areas for the High condition at 300–400 ms, while it was not clearly observable in the Low condition. The ERP amplitude in this epoch was significantly reduced for the Low condition as compared to the High condition (green-shaded epoch in Figure 4a, paired *t*-test, *p* < 0.001).

Figure 4c,d show the waveforms at the parietal region (average of P3, Pz, and P4) and topographies (at 515 ms) of the second ERP components, respectively. Notably, the latency and topography of the second component were observed to be similar to those for the sudden stop task shown in Figure 2c,d. That is, a broad and widespread positive peak was observed over the parieto-occipital region at 450–700 ms, as shown in Figure 4c,d. Moreover, the amplitude was significantly decreased for the Low as compared to the High vigilance condition in the 450–700 ms epoch (paired *t*-test, *p* < 0.001), just as for the sudden stop task.

**Figure 5:**
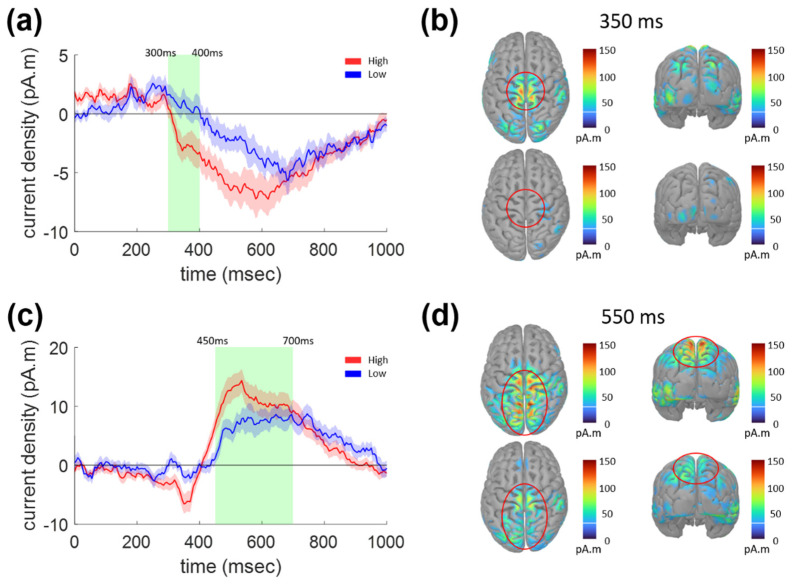
ERCD waveforms for the lane departure task (0–1000 ms) and the distribution of current source density on cortical surface. (**a**) ERCD waveforms averaged over the vertices belonging to postcentral regions. (**b**) Distribution of ERCD on cortical surface at 350 ms. (**c**) ERCD waveforms averaged over the vertices belonging to superior parietal regions. (**d**) Distribution of ERCD on cortical surface at 550 ms.

The two ERP components for the lane departure task could also be characterized from the estimated ERCD time series shown in Figure 5a,c, respectively. The ERCD amplitude averaged for the vertices within the postcentral region in the Desikan-Killiany atlas was significantly different between the two vigilance conditions during the epoch of the first ERP component, 300–400 ms, as shown in Figure 5a (paired *t*-test, *p* < 0.014). The ERCD peaked around the central sulcus, as denoted by red circles in Figure 5b, whereas it was absent in the Low condition.

Figure 5c,d show the temporal and spatial characteristics of the ERCD, which was expected to underlie the second ERP component with 450–700 ms latency. High ERCD was focused within the superior parietal and central regions, as denoted by red circles in Figure 5d, which was very similar to the case for the sudden stop task illustrated above (shown in Figure 3c,d). The ERCD amplitude averaged for the vertices within the superior parietal area in the Desikan-Killiany atlas was significantly smaller for the Low as compared to the High vigilance condition (paired *t*-test, *p* = 0.0154). Once again, this change in ERCD amplitude according to the vigilance condition was identical for the two tasks.

## 4. Discussion

In this study, we investigated the spatiotemporal characteristics of brain activity while unexpected events occurred during monotonous driving. Two types of unexpected events were presented: (1) sudden stop of the car just in front, and (2) lane departure due to unexpected car movement against the driver’s will. These two types of events were expected to be recognized by means of visual and somatosensory/vestibular sensation and result in behavioral responses, by pushing the brake pedal or turning the steering wheel. We found that common characteristic brain activities occurred in response to sudden events, regardless of the type. Moreover, the modulation of brain activity due to the vigilance level also shared common characteristics between the two types.

From the ERPs of both types of events, an early component representing sensory information processing and a late component were observed. The early ERP component could be interpreted as reflecting visual and somatosensory/vestibular information processing for the sudden stop and lane departure tasks, respectively. The late ERP components showed spatiotemporal characteristics of the well-known P300 component for both types of events.

In terms of methodology, the advantage derived from the use of simultaneous analyses of scalp sensor-level waveforms and cortical current source densities. This enabled us to compare our results with previous studies, which were based on sensor-level ERP analysis, and to identify the cortical origin of the ERP components. Thus, we could suggest possible explanations for the similarity and difference between the two modalities.

### 4.1. Early Visual Component

We found a prominent occipital ERP component at 200–325 ms, with a significant negative peak at ~300 ms for the sudden stop task. Source analysis confirmed the activation of the occipital visual area, which was significantly higher in the high vigilance condition. The bilateral occipital area is known to be involved in the cognitive selection of sensory information and responses as part of visual attention [10]; specifically, it was found to be responsible for visual event detection during driving tasks [11,12,13]. Previous driving simulation studies have shown that brain activity for low-level visual processing is reflected in an ERP component in the occipital region during 200–350 ms [14,15,16].

From the spatiotemporal characteristics of the brain activity observed during the early epoch of a sudden stop, we expect that the early bilateral occipital activity reflects a process of visual information processing caused by the tail light flash of the preceding vehicle. In addition, the decrease in this activity for the low vigilance condition is judged to result from a decrease in visual attention. This is in line with a previous study that observed that occipital activity during driving was reduced for higher cognitive load [17].

### 4.2. Early Somatosensory/Vestibular Component

For the lane departure task, a strong negative peak in the ERP waveform was found at ~360 ms, focused within the midline centro-parietal regions for the High condition. However, it was not possible to find this ERP component for the Low condition. The driver must predict rapidly how the vehicle will move and prepare to return to the original lane position when lane departure occurs. We interpret the absence of the early ERP peak for the Low condition as being due to the ineffective cognitive capability to perceive the information of the slipping and rotating, resulting in a significantly delayed and jittered response.

The origin of this ERP component was found to be located in the somatosensory and motor areas. Dominant sources were distributed over the bilateral superior parietal regions and around the central sulcus. Although the stimulus for the lane departure situation may incorporate visual sensation, we estimate that somatosensory activation, vestibular activation, or a complex of these should play critical roles in detecting lane departure.

The characteristics of ERP under lane departure due to car-drifting are not as well-known as the visual ERP for the sudden stop task. However, it is known that parts of the motor area are activated not only when performing motor activation, but also when observing movement [18,19,20]. It was also reported that the driver’s somatosensory area is activated to perceive movements by means of spatial cues such as position, direction, and speed, which change continuously, in a lane departure situation [20]. This may reflect the process of automatic activation of the motor system due to prior knowledge of the upcoming action in preparation [19]. This activity is known to be reflected in a slow negativity around the central region observed from ~1.5 s before spontaneous movement occurs. It is known that negativity increases continuously until movement onset [19]. The car drifting stimulation presented in this study could also be interpreted as transient horizontal body rotation, which triggers vestibular stimulation. It has been reported that vestibular stimulation is observed not only when the body is actually moved but also by visual movements such as optokinetic stimuli [18,19].

### 4.3. P300 Component

A large and broad parietal ERP component was observed at 450–700 ms for both types of sudden events. A positive peak was found at ~500 ms for both types, and interestingly, its amplitude was reduced in the Low condition for both types. Source analysis showed the origin of the ERCD over the bilateral superior parietal regions and around the central sulcus. Based on the spatiotemporal characteristics, we expect that this activity corresponds to the well-known P300, which is known to be observed from ERP waveforms in a variety of sudden event detection tasks. It can be inferred that diminished attentional capability and inefficient cognitive processing resulted in impaired driving performance in the Low vigilance condition. The P300 amplitude is known to be reduced as concentration decreased due to mental fatigue [21], and also because of the increased task difficulty. This is expected to originate from inefficient sensory processing or dysfunction of attention maintenance [22,23,24], resulting in a higher demand for attention resources required for updating contextual information in working memory [25,26].

### 4.4. Response-Related Activities

After 400 ms, as shown in Figure 3d and Figure 5d, dominant cortical activities were distributed over the precentral, postcentral, and parts of the superior frontal regions. The superior frontal activity is thought to be caused by the frontoparietal network underlying the P300 component. In previous studies, superior frontal activities were observed along with superior parietal activities for goal-directed movements involving stimuli-driven attentional networks [10,23], which may involve pre- and post-central activities for movement prediction and motor response generation [14,15,16].

### 4.5. Application and Further Studies

The result of this study may be applied to developing a human-machine interface for automated driving assistance systems [27,28]. The emergency events may be detected based on the features of ERP waveforms, which were characterized in this study. This should be useful for practical situations where the driver’s attention level is low. However, further study is necessary to implement a pattern recognition system that provides reasonable performance using single-trial ERP waveforms. Moreover, the EEG waveforms should be acquired from an easily wearable sensor module, preferably with few channels [2,27,29].

EEG source localization is known to suffer from an inherent limitation of spatial resolution and absence of unique solution. The cortical origin of the neural activities should be further verified using functional neuroimaging with better spatial accuracy, such as functional magnetic resonance imaging.

## 5. Conclusion

We investigated the spatiotemporal characteristics of brain activity due to sudden events during driving and their changes due to vigilance level, based on an ERP study. Regardless of the types of sudden events, an early component representing sensory information processing and a P300-like component were observed. The vigilance level similarly affected the brain activity for the two types of tasks. Our results may be applied to develop a human-machine interface for an automated driving assistance system for emergency situations. Further study is necessary to enable emergency detection based on single-trial ERP with few channels.

## Data Availability

The data are not publicly available due to privacy issues. The data presented in this study are available upon request from the corresponding author. If necessary, please contact the author.
